# Development and Testing of a Novel Arm Cranking-Powered Watercraft

**DOI:** 10.3389/fphys.2017.00635

**Published:** 2017-08-29

**Authors:** Thomas Fuglsang, Johnny Padulo, Massimo Spoladore, Michele Dalla Piazza, Luca P. Ardigò

**Affiliations:** ^1^Department of Neurosciences, Biomedicine and Movement Sciences, School of Exercise and Sport Science, University of Verona Verona, Italy; ^2^Sport Science, University eCampus Novedrate, Italy; ^3^Faculty of Kinesiology, University of Split Split, Croatia; ^4^Research Laboratory “Sport Performance Optimization”, National Center of Medicine and Sciences in Sport Tunis, Tunisia

**Keywords:** efficiency, human-powered boats, metabolic cost, metabolic expenditure, spinal cord injury

## Abstract

There is a lack of human-powered watercrafts for people with lower-body disabilities. The purpose of this study was therefore to develop a watercraft for disabled people and investigate the metabolic cost and efficiency when pedaling. The watercraft was designed by combining parts of a waterbike and a handbike. Nine able-bodied subjects pedaled the watercraft at different speeds on a lake to provide steady-state metabolic measurements, and a deceleration test was performed to measure the hydrodynamic resistance of the watercraft. The results showed a linear correlation between metabolic power and mechanical power (*r*^2^ = 0.93). Metabolic expenditure when pedaling the watercraft was similar to other physical activities performed by people with lower-body disabilities. Moreover, the efficiency of the watercraft showed to be comparable to other human-powered watercraft and could, as a result, be an alternative fitness tool especially for people with lower-body disabilities, who seek water activities. A number of suggestions are proposed however, to improve the efficiency and ergonomics of the watercraft.

## Introduction

Spinal cord injury (SCI) may impair sensory, motor, and autonomic function below the level of the injury. As a result, people suffering from SCI often develop secondary impairments, such as cardiovascular diseases, pressure ulcers, and musculoskeletal pain. These impairments are often the consequence of a sedentary lifestyle in a wheelchair, a device that 80–90% of persons with SCI rely on in everyday life (Biering-Sørensen et al., 2005). People with SCI are among the most inactive ones in our society (Fernhall et al., 2008), as 50% of people with SCI reported <30 min of mild intensity activity *per* day in a study, which was considered insufficient to maintain or improve physical capacity by the authors (Gibbons et al., 2014). Accordingly, additional physical activity is recommended for wheelchair users as physical activity is related to a reduced risk of cardiovascular diseases (Phillips et al., 1998), and obesity (Buchholz and Pencharz, 2004), and leads to less pain and fatigue in everyday life (Tawashy et al., 2009).

A popular form of physical activity for SCI persons is handcycling. Handbikes for handcycling are driven by an arm crank system, similarly to what is known in cycling, but they are usually equipped with synchronous hand-pedals, three wheels, and a seat. Handbikes are more mechanically efficient than everyday wheelchairs, and handcycling is highly recommended to maintain the level of physical fitness and to prevent atherosclerosis, as it is characterized by a relatively high-energy consumption at moderate training intensities (Abel et al., 2006). Furthermore, the closed-chain motion of handcycling enables propulsion force to be applied throughout the whole 360° of crank rotation, which is suggested to cause less musculoskeletal strain compared to everyday wheelchair use, and thereby decrease the risk of overuse injury (Dallmeijer et al., 2004b).

People suffering from SCI also have the option of practicing both alpine and cross-country skiing (using sit skis), which assist the para-skiers with balancing, turning, and controlling the speed. Equipment also exists that enables SCI people to participate in activities, such as basketball, rugby, tennis, and throwing events. At present, however, it is difficult especially for people with high-to-medium level (HML) SCI to navigate with human-powered watercrafts, such as rowing boats, canoes, and kayaks, mainly due to the trunk movement impairment that often follows SCI. Accordingly, a new human-powered watercraft that is suitable for people with HML SCI would represent a highly valuable option for them for fitness and leisure activities as well. The aim of this study was to develop such a watercraft and investigate the metabolic cost and efficiency when pedaling the watercraft, using correlational analysis.

## Materials and methods

### Subjects

The experiments were carried out on nine (eight males and one female) able-bodied subjects (34.2 ± 8.3 years; 75.3 ± 9.5 kg; 1.75 ± 0.07 m). The subjects had no experience in handcycling or injuries of the upper extremities. All subjects gave their written informed consent before testing, and were thoroughly informed about the purpose, benefits, and potential risks of the study, in conformity with the Code of Ethics of the World Medical Association (Declaration of Helsinki). The protocol and the methods applied in the study were approved by the Ethical Committee of the Department of Neurosciences, Biomedicine and Movement Sciences, University of Verona.

### Watercraft

**Figure 1:**
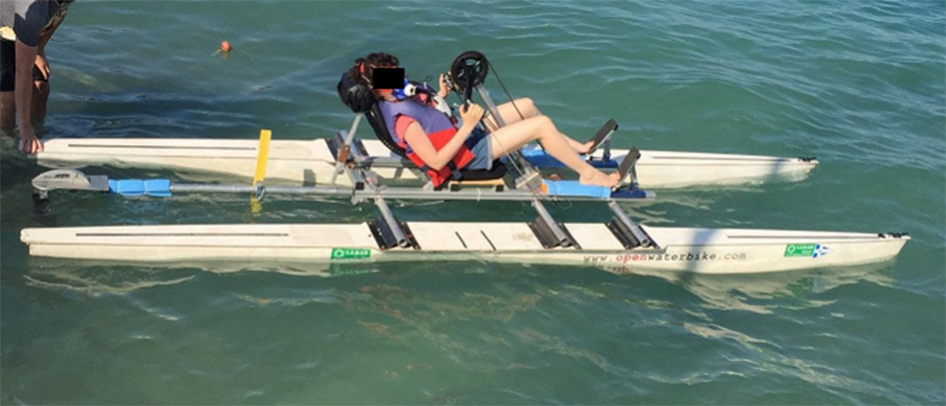
The Handwaterbike.

The watercraft, named the Handwaterbike, is a catamaran consisting of two carbon hulls to provide buoyancy, whereas a recumbent handbike seat (Maddiline Cycle, Sant'Ambrogio di Valpolicella, Italy), and footrests are fixed between the two hulls by means of a custom-built aluminum frame (Figure 1). The frame is placed on top of three aluminum pipes, which are tightened to the hulls using screws. The seat is attached to a wooden plate, which is attached to the frame using angular fittings. A synchronous arm-crank system in front of the seat is connected *via* a roller chain to the transmission system, which drives a flexible shaft and the propeller. The ratio of the chain ring to the sprocket is 52:15 or 3.47. The propeller has a diameter of 330 mm and a pitch of 450 mm. When pedaling is paused, the propeller folds together, removing potential seaweed from the blades. The rudder is positioned rear of the seat, and is connected to the gear-shifters on the left and right handle (Maddiline Cycle, Sant'Ambrogio di Valpolicella, Italy) *via* a wire. Because the wire is in tension, the rudder turns either left or right when one of the gear-shifters is pushed, depending on which gear-shifter is pushed. The more it is pushed, the more the rudder turns. This system allows the user to keep pedaling even when maneuvering the boat. The main dimensions of the Handwaterbike are: length over all: 4.89 m; length of water line: 4.77 m; weight without subjects: 69.17 kg; maximal beam: 1.08 m; draft (the vertical distance between the waterline and the bottom of the hull): 0.10 m.

### Measurements

**Figure 2:**
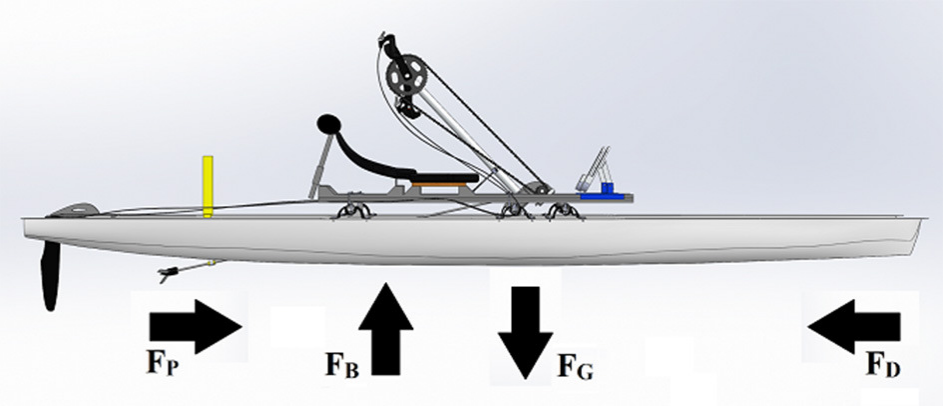
3D CAD model of the Handwaterbike and a free body diagram of the forces acting on the system. F_P_, propulsive force; F_B_, buoyant force; F_G_, gravitational force; F_D_, drag force.

Oxygen consumption ($\dot{V}O2,\text{ L }\cdot \overset{-1}{\min}$), carbon dioxide production ($\dot{V}CO_{2},\text{ L }\cdot \overset{-1}{\min}$), and heart rate (HR, bpm) were assessed breath-by-breath using a portable metabolic system (K5, COSMED, Rome, Italy). Before each test, the system was calibrated according to the manufacturer's instructions. The Handwaterbike was instrumented with a power-meter crankset (Quarq RIKEN R, SRAM, Spearfish, SD, USA) with a crank length of 170 mm allowing the measurement of pedaling frequency (rev·$\overset{-1}{\min}$) and of external mechanical power (Ẇ) at a sampling rate of 60 Hz. Boat speed was measured at a sampling rate of 1 Hz by means of a GPS receiver (Rider 20, Bryton Inc., Taipei City, Taiwan) fastened to the arm-crank system. A 3D computer-aided design model of the Handwaterbike (Figure 2) was created using a CAD software (SolidWorks, Dassault Systèmes SolidWorks Corporation, Waltham, MA, USA) in order to measure the frontal surface area of the submerged part. The submerged area was corrected by accounting for the additional volume of water displaced when a subject was on board.

### Experimental protocol

The experiments were performed along the shore of the Garda Lake (Italy) in basically calm water and with wind speed always <2 m · s^−1^. The subjects were asked to pedal in a linear direction for at least 5 min, to allow steady-state metabolic measurements, and at three different constant speeds: (1) a speed the subject would be able to maintain for approximately 1 h, (2) a “little bit slower than that,” and (3) a “little bit faster than that.” Before the trials, the subjects were asked to sit on a chair for 5 min. for measurement of metabolic variables at rest. Before the subjects could begin a new trial, they had to rest for at least 5 min, have a heart rate below 100 bpm, and communicate their availability to re-start.

A deceleration test was also performed to measure the hydrodynamic resistance of the Handwaterbike. A subject was instructed to pedal the boat at a constant speed of 2.2–2.8 m·s^−1^ before letting go of the handles. This procedure was repeated along the same segment six times, three times *per* direction to account for the influence of water stream, and overall average result was used. The speed of the Handwaterbike was measured by the GPS receiver during deceleration and using the method described by Bilo and Nachtigall (1980), and Capelli et al. (2009). The boat drag was calculated by analyzing the time course of the decreasing speed as a function of time. Data recorded for the first approximately 10 s during the deceleration tests were used for calculation of the hydrodynamic resistance.

Finally, the relationship between mechanical power output and boat speed was investigated by increasing the power by 10 W every 10 s until reaching 120 W. This was done three times in each direction to account for the influence of water stream, and overall average result was used.

### Data analysis

Only data collected from the last minute of each trial was used for analysis. Coefficient of variation (CV) of oxygen consumption data for the last minute of each trial was compared to the preceding minute of each trial to ensure steady-state was reached. All mean CV was <10%, which indicates that steady-state was reached. Mean oxygen consumption was converted into metabolic power using the empirical formula $Ė=\left(\left[4.94\;\times \text{ RER}+16.04\right]\;\times \;\dot{V}O2/60\right)$ (Garby and Astrup, 1987), where RER is respiratory exchange ratio, and $\dot{V}O2$ is the net oxygen uptake (above that measured at rest). The metabolic power was then divided by the average speed during the corresponding trial to calculate the metabolic cost of locomotion, C. The water drag (D) was calculated using the formula: $D=C_{D}\;\times \;A\;\times \;\rho \;\times \;v^{2}\;/\;2$ (Capelli et al., 2009).

Metabolic equivalent (MET) was calculated by dividing the oxygen consumption by 3.5 mL^−1^ · kg^−^1 · min^−1^ (Ainsworth et al., 2011). The methods of Zamparo et al. (2008) were used to calculate power to overcome hydrodynamic resistance Ẇ_*d*_, net mechanical efficiency η_0_, propelling efficiency η_*p*_, and drag efficiency η_*d*_.

As descriptive statistics, mean values and standard deviations were used. Data from the metabolic system were low-pass filtered (2nd order Butterworth with a cutoff frequency of 0.1). The Pearson correlation coefficient (*r*) was used for correlation analysis. For inferential statistics purpose, level of significance was set at *P* < 0.05. Coefficient of determination (*r*^2^) was calculated to assess the strength of *r*. Statistical analysis was carried out using Microsoft Excel (Microsoft, Redmond, Washington, USA).

## Results

**Figure 3:**
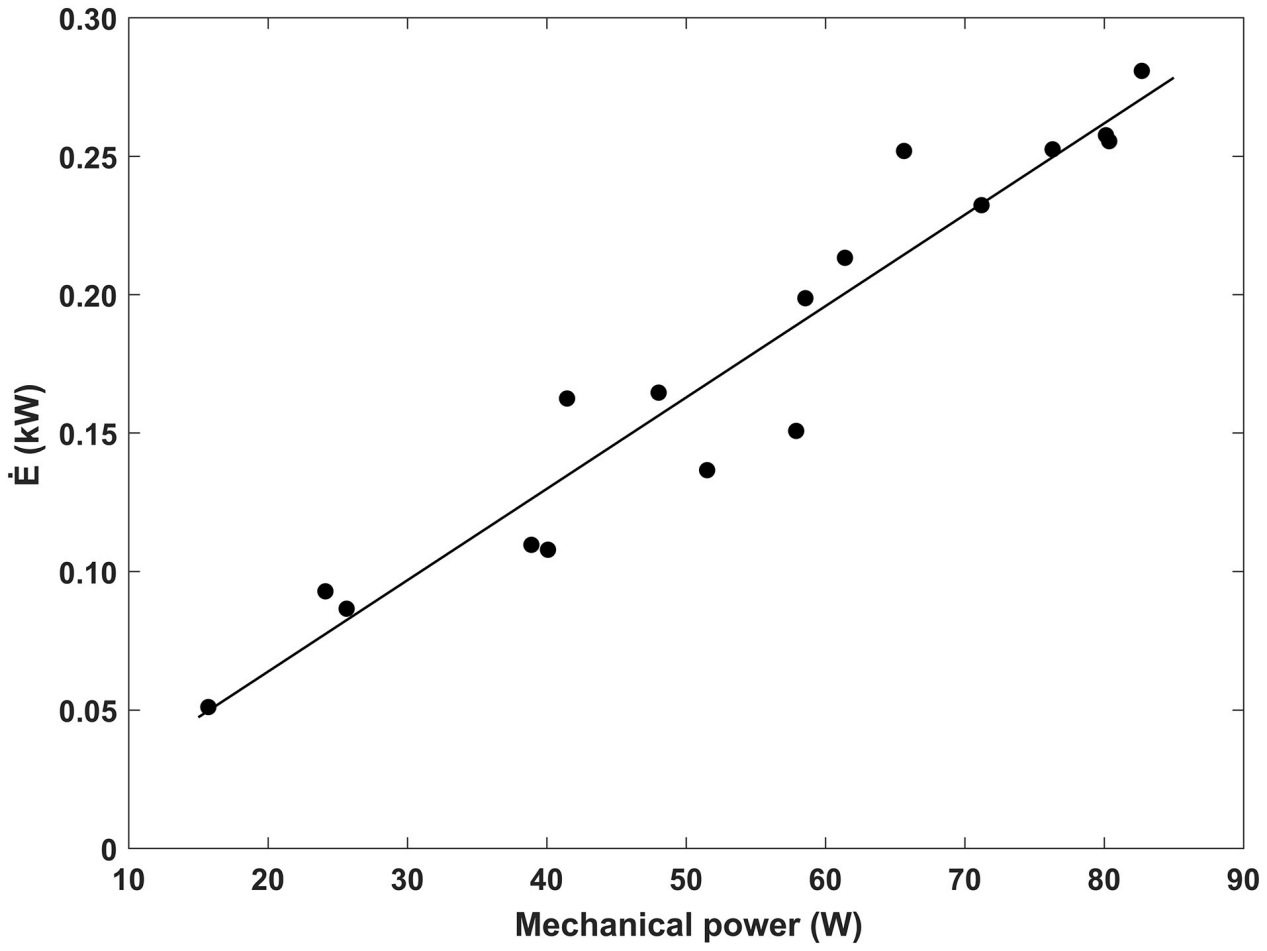
Metabolic power (Ė) at steady state plotted as a function of the mechanical power (Ẇ) while pedaling the Handwaterbike.

**Figure 4:**
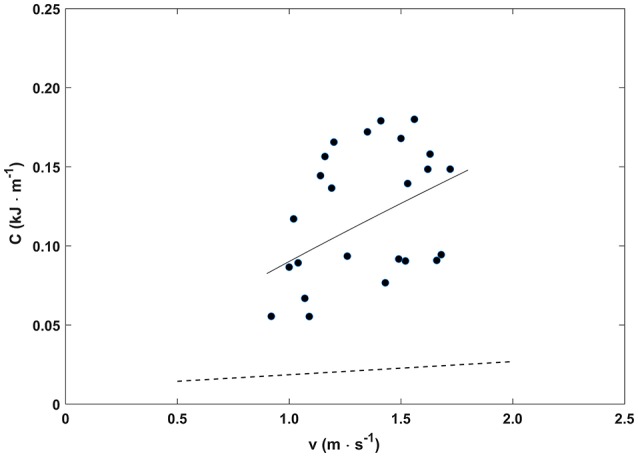
The metabolic cost needed to cover one unit distance (C) plotted as a function of the speed (υ) for the Handwaterbike (continuous line) and for handbiking (dotted line) (Capelli et al., 2008).

In Figure 3, the metabolic power at steady state Ė (kW) during pedaling the Handwaterbike is plotted as a function of mechanical power (Ẇ). Data were fitted by a linear function Ė = 0.0033·Ẇ−0.0022; *r*^2^ = 0.93; *SEE* = 0.02; *P* < 0.005. The metabolic cost of locomotion C (kJ · m^−1^) for pedaling the Handwaterbike as a function of the speed *v* (m·s^−1^) is shown in Figure 4 and fitted by a (even if not significant) power function *C* = 0.0901·υ^0.84^; *r*^2^ = 0.19;*SEE* = 0.04; *P* > 0.05.

**Figure 5:**
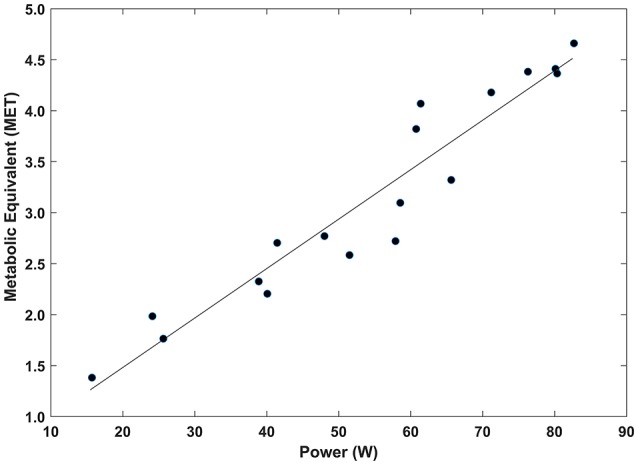
The metabolic equivalent (MET, where 1 MET is defined as the oxygen cost of sitting calmly, equivalent to 3.5 ml/kg/min) plotted as a function of the mechanical power (Ẇ).

In Figure 5, the metabolic equivalent (MET) during pedaling the Handwaterbike is plotted as a function of mechanical power (Ẇ). Data were fitted by a linear function metabolic equivalent = 0.0485·Ẇ+0.5105; *r*^2^ = 0.92;*SEE* = 0.31; *P* < 0.005.

**Figure 6:**
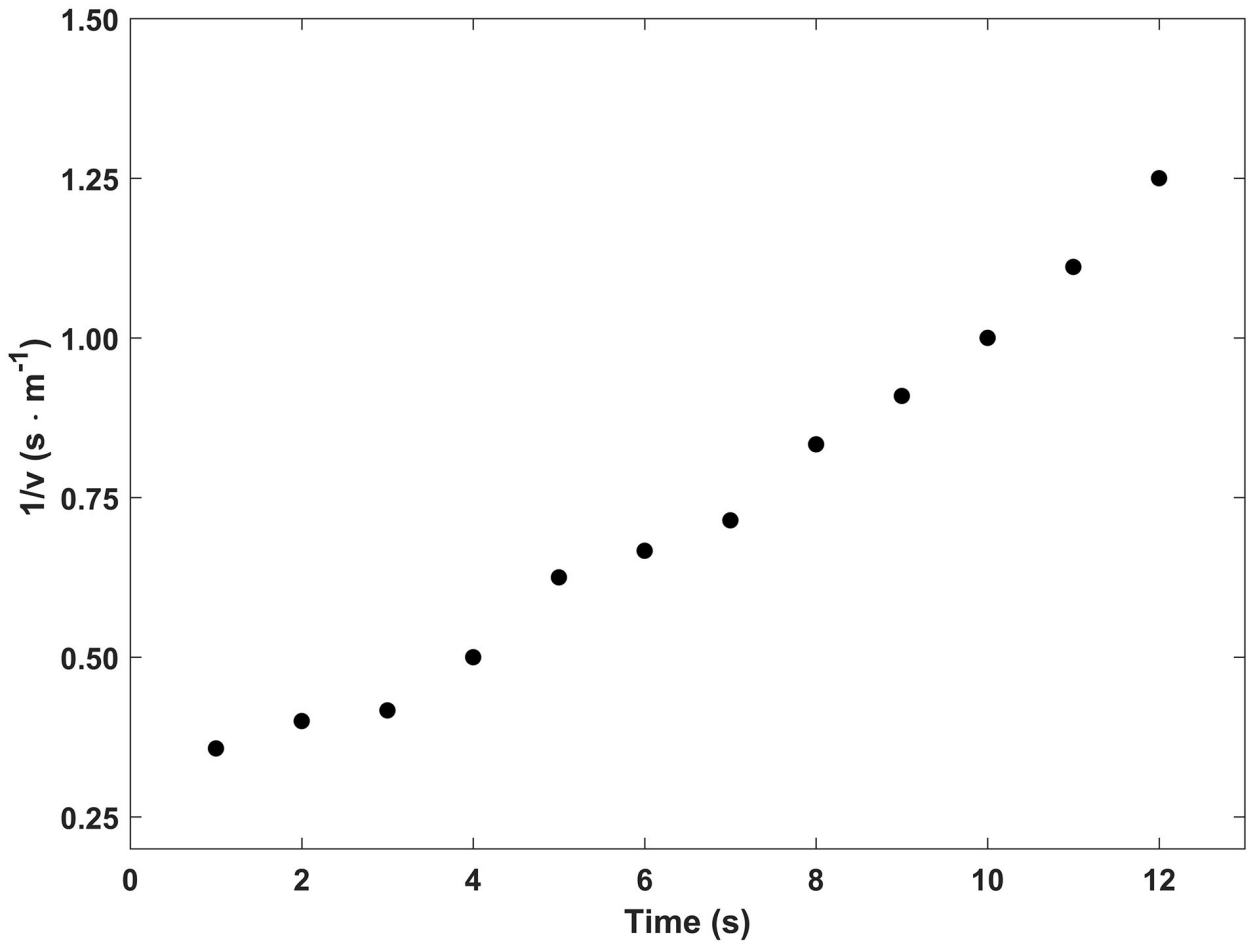
The reciprocal of the decreasing speed (υ) obtained during a typical experiment of spontaneous deceleration plotted as a function of the time.

In Figure 6, the reciprocal of the decreasing speed, recorded during one of the deceleration trials, is shown as a function of time. The average slope of the linear regressions calculated from the six trials was 0.082 ± 0.015 s · m^−1^. With this value and the overall mass of the boat, the maximal frontal submerged area and the water density, the dimensionless *C*_*D*_ was calculated as 0.427 using the method described by Capelli et al. (2009). Knowing this, water drag of the Handwaterbike could be described by the following equation: *D* = 5.64·υ^2^.

Net mechanical efficiency (η_0_) when pedaling the Handwaterbike was 0.29 ± 0.03 and was independent of both mechanical power output: $\eta_{0}=0.0001\cdot W+0.2906;r^{2}=0.0079;\;P>0.05$, and cadence (RPM): $\eta_{0}=0.00007\cdot RPM+0.3033;r^{2}=0.00009;\;P>0.05$.

## Discussion

The aim of this study was to investigate efficiency and metabolic cost of pedaling a novel human-powered watercraft for people with lower-body disabilities. The data shows a strong linear correlation between the metabolic power and the mechanical power. The subjects could reach mechanical power outputs above 80 W. Arm cranking at this power has been shown to be sufficient for maintaining or improving cardiovascular health and fitness, and likely help prevent cardiovascular diseases for people with SCI (Abel et al., 2003a). The Handwaterbike has a single gear ratio and the testing demonstrated that this was sufficient at power outputs from 20 to 80 W. Experienced handcyclists, however, may be able to produce a significantly higher power output, resulting in a higher cadence, which at some point will be impossible to increase. This could limit the utilization of the Handwaterbike as a training tool for this population. Another potential problem relates to propeller ventilation, which occurs when air is drawn into the water flowing to the propeller (Kozlowska et al., 2009). The most common cause of propeller ventilation is when waves cause the propeller blades to break the water surface and become exposed to the air. Propeller ventilation can also occur during rapid accelerations. Then, if the spinning of the propeller is sufficiently fast, it causes a vortex from the surface to blades. Ventilation leads to sudden large losses of propeller thrust and torque, and could damage the propeller.

Several studies have demonstrated that additional energy consumption due to physical activity decreases the risk of mortality and morbidity (Blair and Connelly, 1996; Blair and Brodney, 1999). Figure 5 shows a linear relationship between metabolic equivalent and mechanical power output. At a moderate mechanical intensity of 50 W, the corresponding metabolic power is 2.9 MET. This is similar to activities, such as wheeling outside, weight training and circuit training (Collins et al., 2009). Tweedy et al. (2017) recommend people with SCI to do ≥30 min of moderate exercise, defined as 3–6 METs, at least 5 days *per* week.

The metabolic cost of locomotion and speed showed a very low correlation with each other. This is likely due to the multiple testing days. Even though the wind speed was lower than 2 m ·s^−1^ during all trials, it is plausible to think that some light water streams may have influenced the boat speed. If the testing had been performed during the same day or in a pool, the correlation would presumably have been higher at the cost of a lower ecological setting.

As indicated in Table 1, where all data refer to a metabolic power (Ė) of 0.5 kW, the Handwaterbike reaches similar speeds to other reported boats in the literature, such as a waterbike and a rowing shell, but is faster than a paddle-wheel boat and a slalom kayak. The power to overcome drag (Ẇ_*d*_) for the Handwaterbike is similar to the waterbike but lower than for the slalom kayak and rowing shell. Consequently, the same is true for the drag efficiency (η_*d*_) since it is calculated as $\eta_{d}=\frac{{Ẇ}_{d}}{Ė}$. η_*d*_ is defined as the efficiency with which the metabolic power is transformed into useful mechanical power (Zamparo et al., 2008). This is to be expected since the waterbike and the Handwaterbike are catamarans with two symmetric hulls, whereas the kayak and the rowing shell are monohull boats and therefore likely with a smaller submerged frontal area. Lowering the weight of the relatively heavy aluminum frame could reduce the submerged frontal area of the Handwaterbike. It has to be noted though that the Handwaterbike is mainly intended for use by people with lower-body disabilities. Consequently, a high level of stability is needed, even if it negatively influences the performance of the watercraft. Similar to how a handbike worsens its performance by having three wheels instead of two (Fischer et al., 2014, 2015). Some boats are able to maintain a low drag and high stability by having a long keel that provides lateral resistance to prevent the wind or waves from pushing the boat sideways. This solution however, can be problematic as the long keel makes it difficult for the boat to access smaller ports and to travel along the beach. Instead, a catamaran is able to achieve a good level of stability, low draft, and relatively low drag by using two hulls but thus increasing the width of the watercraft.

**Table 1:** Comparison of five boats at a metabolic power (Ė) of 0.5 k W (adapted from Zamparo et al., 2008).

	**Paddle-wheel boat**	**Slalom kayak**	**Water bike**	**Rowing shell**	**Handwaterbike**
υ (m·s^−1^)	1.3	1.8	2.3	2.4	2.4
Ẇ_*d*_ (*W*)	44	85	73	99	74
Ẇ_*tot*_ (*W*)	127	122	128	141	152
η_*p*_	0.39	0.70	0.57	0.70	0.49
η_0_	0.27	0.24	0.27	0.27	0.29
η_*d*_	0.09	0.17	0.14	0.19	0.15

*υ speed, Ẇ_d_ power to overcome hydrodynamic resistance, Ẇ_tot_ total power output, η_p_ propelling efficiency, η_0_ overall efficiency, η_d_ drag efficiency*.

Table 1 shows that at an Ė of 0.5 kW the corresponding mechanical power output would be 152 W for the Handwaterbike. This is comparable, even if slightly higher, than a paddle-wheel boat (127 W), slalom kayak (122 W), water bike (128 W), and a rowing shell (141 W). This is likely due to the different propulsion styles of the boats. Propulsion is generated for the paddle-wheel boat and the water bike by leg pedaling in a semi-recumbent position, whereas propulsion for the rowing shell is generated by leg drive and arm pull of the oars. Propulsion of the slalom kayak is generated by an upper hand push and lower hand pull of a paddle, supported by a concomitant rotation of the torso. It should be noted though that Table 1 refers to a comparison between all the watercrafts at an Ė of 0.5 kW and even though Figure 3 shows a linear relationship between Handwaterbike Ė and W, we only have data up to 0.3 kW in the present study. It cannot be excluded that the relationship will look different at higher power outputs.

The Handwaterbike has a net mechanical efficiency (η_0_) similar to the other boats in Table 1. η_0_ is also similar to that measured with cycle ergometers (Zamparo et al., 2008). This is somewhat surprising since η_0_ of arm exercise has been reported to be lower than that of leg exercise (Marais et al., 2002), probably due to the smaller and different (in terms of fiber types content) muscle mass involved in arm cranking vs. cycling. An explanation could be that the subjects in this study were able-bodied and able (or freely self-compelled) to use all of their upper body to create propulsion and not just their arms. Especially people with high-level SCI are likely to have a lower η_0_ when pedaling the Handwaterbike. It was expected that able-bodied subjects would respond relatively homogenously to pedaling the Handwaterbike, since they were equally inexperienced in arm cranking and had no restriction due to disability. It should also be noted that energy use is likely lower in persons with SCI compared to abled-bodied persons due to decreased fat-free mass and sympathetic nervous system activity (Buchholz and Pencharz, 2004). As a higher level of SCI has been shown to correlate with lower energy use (Abel et al., 2008; Collins et al., 2009), it is likely that the energy use would be lower if the Handwaterbike were propelled by SCI people as well. Net mechanical efficiency (η_0_) showed to be independent of mechanical power output and cadence. This is similar to Goosey-Tolfrey et al. (2008), who found no significant difference in mechanical efficiency between arm cranking at 70 RPM and 85 RPM. This is in contrast to cycling, where the mechanical efficiency tends to decrease when cadence is increased (Sacchetti et al., 2010). Reservations should to be made, however, toward analyzing the cadence in our study since the Handwaterbike was limited to a single gear ratio. As a result, cadence will have been greatly influenced by especially stream direction and magnitude. Propelling efficiency (η_*p*_), defined as the ratio of useful work to total work production, was lower for the Handwaterbike than all other boats in Table 1 except for the paddle-wheel boat, which was expected to be less efficient, since energy losses for paddle-wheel system has shown to be quite large (Zamparo et al., 2008). The efficiency of the customized propeller mounted on the Handwaterbike varies according to the loading condition, but should be around 78%, when used on the Handwaterbike, according to the manufacturer. The remaining loss of efficiency is due to friction in the transmission chain, especially in the bearings and the roller chain.

As seen in Figure 2, the watercraft is affected by four forces: gravity, buoyancy, propulsion and drag. At (floating) constant speeds, the gravity and buoyancy forces are equal, and the propulsion and drag forces are equal as well. The drag forces consist of air and hydrodynamic drag. The hydrodynamic drag is dominant for most watercrafts as air density is much lower than water density, and air drag only contributes to 10% of the total drag for a rowing system (Baudouin and Hawkins, 2002). The hydrodynamic drag consists of form drag, skin drag, and wave drag. Form drag depends on the shape of the watercraft and hulls, such as the one used in this study already approach the optimal shape for drag considerations (Baudouin and Hawkins, 2002). Skin drag depends on the friction that occurs between the water and the hull, and is responsible for over 80% of the hydrodynamic drag on a racing hull (Baudouin and Hawkins, 2002). A boundary layer is created when a thin layer of water is accelerated to the speed of the hull (Buckmann and Harris, 2014). The Reynolds number, i.e., the ratio of inertial forces to viscous forces, determine whether the boundary layer is laminar or turbulent. Laminar flow is a smooth and steady flow, whereas turbulent flow is a chaotic and unsteady flow. At the hull's bow, the boundary layer is laminar but it quickly starts turning turbulent. In fact, approximately 80% of the hull is in a transitional flow from laminar to turbulent (Pendergast et al., 2005). Skin drag can be decreased if such a transition is delayed and the laminar area is increased, since laminar flow produces less skin friction than turbulent flow (Day et al., 2011). It has been suggested that skin drag could be reduced by applying a hydrophobic coating on the surface of the hull (Baudouin and Hawkins, 2002). Another skin drag possibility could be to alter the texture of the wetted hull surface. A smooth surface does not always result in minimal skin drag, as researches, inspired by the riblet surface of shark skin, have shown (Dean and Bhushan, 2010). Another skin drag reduction example is how dimples reduce the drag on a golf ball. Separation bubbles with small distinctive dimensions are generated inside the dimples resulting in low Reynolds numbers (Choi et al., 2006). A further and obvious way to reduce skin drag is to reduce the weight of the system, thereby reducing the wetted area. As mentioned earlier however, this could negatively impact the stability of the Handwaterbike. The last form of hydrodynamic drag, wave drag, occurs as a result of pushing the water away from the hull. The hulls in the present study have bows with sharp angles, thereby pushing the water away gradually. As a result, wave drag is not a significant source of drag for long thin hulls (Baudouin and Hawkins, 2002).

As previously mentioned, the arm-crank system for the Handwaterbike is synchronous. Whereas, this system has shown to be more efficient than an asynchronous arm-crank system in handbiking (Abel et al., 2003b; Dallmeijer et al., 2004a; Bafghi et al., 2008), the applied tangential force is not constant through the entire crank revolution, but peaks during the pull down and push up phase (Arnet et al., 2012). On a watercraft, this could result in fluctuations of boat speed. Water resistance is increased approximately four times when speed is doubled (Hill and Fahrig, 2009). Therefore, it would be most effective to keep the speed of the watercraft as constant as possible. When pedaling the Handwaterbike, an asynchronous arm-crank system should provide a more constant power output and might lead to the result of a higher efficiency. This should be tested in the future by comparing the metabolic cost of synchronous and asynchronous arm-cranking on the Handwaterbike at similar mechanical power outputs.

Testing highlighted some ergonomic problems with the Handwaterbike, mainly regarding handles, seat, and backrest and footrests adjustability. Every element is adjustable to a certain degree, but it requires specific tools and know-how. A future version of the Handwaterbike should look to make the possibilities for adjusting the watercraft to fit different users easier and faster. As mentioned previously, the subjects in this study were able-bodied. This is a limitation of the study since the Handwaterbike is intended for people with lower-body disabilities, a population likely to have a different metabolic response to propelling the watercraft. Outside of this study, several people with lower-body disabilities have tried the Handwaterbike and a paraplegic man participated in the 12 km long Gardalonga boat race on the Garda Lake in Italy (http://www.gardalonga.it/en), and experienced no problems. In the future however, a similar study should be performed with lower-body disabled subjects. Another limitation was that accuracy and precision of the GPS-receiver were not assessed, but there is already literature supporting its use for boat navigation research (e.g., with 1 Hz units achieving <1% error over >100 s durations (Smith and Hopkins, 2012).

## Conclusions

The Handwaterbike is a novel human-powered watercraft designed for people with lower-body disabilities. This study showed that pedaling the Handwaterbike could be a promising fitness tool for people with lower-body disabilities looking for physical activities on the water. Pedaling the Handwaterbike demonstrated metabolic expenditure and power outputs similar to those thought to be sufficient to maintain or improve cardiovascular health and fitness, and reduce the risk of cardiovascular diseases. Several improvements are proposed however, mainly regarding ergonomics and efficiency.

## Author contributions

TF is the corresponding author and has been responsible for all things related to gathering and analyzing the data. JP has especially been helpful regarding statistics and general advice for the manuscript. MS has been the main engineer of the designing and development of the watercraft. MD has been helpful with data gathering and his knowledge of cycling has been helpful during the development as well. LA is the one who first came up with the idea behind the manuscript and has been involved in all things from the first design to the last sentence.

### Conflict of interest statement

The authors declare that the research was conducted in the absence of any commercial or financial relationships that could be construed as a potential conflict of interest.
